# Estimation of unobservable selection effects in on-line surveys through propensity score matching: An application to public acceptance of healthy eating policies

**DOI:** 10.1371/journal.pone.0196020

**Published:** 2018-04-17

**Authors:** Sara Capacci, Mario Mazzocchi, Sergio Brasini

**Affiliations:** Department of Statistical Sciences, University of Bologna, Bologna, Italy; SOAS, University of London, UNITED KINGDOM

## Abstract

The use of model-based propensity scores as matching tools opens the way to the indirect estimation of mode-related measurement effects and selection effects in web surveys, including a component of selection that cannot be traced back to observable characteristics. By matching and comparing respondents from real independent surveys that use the same questionnaire, but different administration modes, it becomes possible to isolate the selection effect induced by unobservable (or unobserved) respondent characteristics. This study applies a stratification matching algorithm to compare a web survey from a proprietary panel with a computer-assisted telephone survey based on random digit-dialing. The experiment is run in two countries (UK and Italy) to check for consistencies across different cultures and different internet penetration rates. The application to the elicitation of support for healthy eating policies indicates large and significant measurement and selection effects. After controlling for differences in the observed characteristics of respondents and the intensity of internet use, findings suggest that web surveys record lower support and higher neutrality. Similarly, after controlling for administration mode and observed respondent characteristics, internet users are less likely to state support compared to non-users. This suggests that unobserved characteristics play a major role, and post-stratification weighting is not a sufficient countermeasure. As demonstrated by the cross-country comparison, rising internet penetration rates are not a guarantee against this type of error, as disparities in these unobserved characteristics are likely to increase at the same time.

## Introduction

The exponential growth of web surveys over the last two decades has sparkled a large body of research aimed at providing taxonomies of the potential sources of bias they are subject to, and tools to assess the quality and reliability of the information they collect [1]. Despite broad consensus about the biases which may be generated by different kinds of web surveys, there is no abundance of empirical studies exploring their extent and nature in a systematic way [2]. Moreover, the existing evidence indicates that the magnitude of these biases varies depending on the survey topic, or even the individual questionnaire item [3]. Even when comparing samples of internet users across different administration modes, there is evidence showing that mode effects vary by item type and age of the respondent [4].

The focus of this study is on stated support to specific health-related public policies, and the selection and measurement effects which undermine its measurement through web surveys. Suggestive evidence on the difference between results from web surveys relative to other survey administration modes has been provided for health-related domains, including quality of healthcare [5, 6], health status and health behaviors [7–12]. Other studies have investigated the influence of web interviews in eliciting public approval of political decisions, with a focus on broader politics, e.g. the evaluation of the economic and security decisions in different US presidential administrations [13, 14], or an assessment of the British decision not to join the Euro [15]. Stephenson [16] targeted more specific policy measures, but still highly related to political views and voting behaviors, considering for example tax cuts, family aid, housing policies, as well as generic healthcare policy.

To our knowledge no structured empirical analysis has evaluated the biases in on-line surveys exploring public support for specific health policy measures. Denscombe [17] provides a comparison between near-identical web and paper questionnaires which include a question on the acceptance of smoking bans from public places, but the study is limited to a sample of 15-year olds from a single school in England.

All of the aforementioned health-related and political studies found significant differences between surveys based on computer-assisted web interviewing (CAWI) and their computer-assisted telephone interviewing (CATI) or face-to-face counterpart. Furthermore, these disparities did not disappear after the application of demographically-related weights aimed at correcting observable unbalances in the composition of the two samples.

Thus, this study provides ex-post empirical evidence on the magnitude and statistical significance of disparities in public support towards healthy eating policies as measured through a CAWI survey on a probabilistic stratified sample extracted from a proprietary panel, relative to a CATI survey based on a random digit-dial (RDD) sampling design.

The challenge of this task lies in the combinations of effects arising from the adoption of different administration modes, sampling frames and sampling strategies. Hence, we propose a strategy based on propensity score matching (PSM), which should be distinguished from the common application of (inverse) propensity scores weighting (PSW) as a post-stratification tool. The latter procedure is effective in reducing the selection bias which can be traced back to the observed characteristics of the respondents, but it may not work for other selection effects driven by unobserved and possibly unobservable characteristics of the respondents [18]. This issue has been shown not only for PSW, but also for other statistical weighting procedures [8, 10, 11, 16, 19]. For example, Couper et al. [20] provide evidence that internet users and non-users do not only differ in terms of demographic characteristics, but also for a range of financial and health-related indicators. Likewise, Duffy et al. [21] show persisting disparities after PSW for items associated with the use of technology (mobile phones, digital television, DVD players, etc.).

## CAWI with proprietary panels vs. CATI-RDD: definitions and classification of errors

In surveys whose focus is on opinions or attitudes there is no objective population benchmark against which one can compare the absolute performance of web-surveys or CATI surveys. In other words, while reference population estimates may exist for variables like age, gender, or other objective characteristics, this is extremely difficult for attitude items and self-reported behaviours, which in addition are strongly influenced by the interview mode [4, 22]. As Couper [1] notes in relation to web surveys, “(we) need to evaluate the quality of a particular approach in light of alternative designs aimed at similar goals”. Thus, in our analysis, we do not focus on the absolute performance of each mode, intended as the difference between the estimated values and the true values [22], but rather on relative differences between the two modes.

In the Methods section we illustrate our error decomposition strategy and the assumptions on which it rests, but first we provide a formal classification of web survey errors based on Couper [1, 3]. The difference between CAWI and CATI estimates can be seen as the combined effect of selection bias, measurement error, and non-response error acting differently on each mode. We follow this classification, and we refer to selection, measurement and non-response *effects* between two modes rather than absolute errors, where the selection effect is defined as the difference in the selection bias between two modes, the measurement effect is the difference in the measurement error, and the non-response effect is the difference between the non-response error.

### Selection effect

Both modes are potentially affected by different selection biases imputable to two main causes: (a) the two sampling frames and the consequent coverage errors are different; (b) non-response rates and errors also differ across the two modes.

Coverage error is due to discrepancies between the target population and the sampling frame from which the sample is extracted [1]. If the population units that are missing from the sampling frame show systematically different characteristics from those included, and these affect the outcome variables, a selection bias occurs. In principle, the CAWI and CATI samples in our study refer to the same population, but the sampling frames are very different. More specifically, individuals may be excluded from the CAWI proprietary panel for three reasons: (a) they have no access to the internet; (b) they have access to the internet but are not reached by the recruitment process; (c) they are reached and have access, but decide not to enter the proprietary panel. Similarly, the sampling frame for the CATI surveys consists in valid phone numbers, hence excluding any individuals with no access to a valid phone number. A second source of discrepancies in selection biases across the two modes is related to non-response patterns. First, non-response rates have been shown to be very different, with telephone interviews recording higher participation [2]. Second, the factors influencing the decision to participate among randomly selected CATI participants are different from those determining the participation of members of a CAWI proprietary panel. The latter strongly depend on the rules regulating panel membership, including the frequency of invitations to participate, the number of required participations, the level of incentives and the opportunity costs of invitees [23].

In this study we had no control or measurement of non-response errors, thus it is not possible to separate its contribution to the overall selection effect from coverage error.

In order to correct for selection bias in surveys, the standard procedure is based on post-stratification, i.e. providing adjustment weights based on observed variables so that the weighted sample matches the target population in terms of these measurable characteristics. Various procedures exist to provide adjustment weights, but they do not lead to a complete elimination of selection biases [19], because selection also occurs on non-observed characteristics which are unrelated or loosely related to the available covariates [20]. The strategy we propose aims at quantifying that component of selection effect which does not stem from objective or observed respondent characteristics, but nevertheless remains a significant source of error after controlling for those variables driving the selection process which can be observed.

### Measurement effect

In a two-mode CAWI-CATI comparison, the measurement effect relates to the fact that the same respondent would give a different answer to the same question, depending on the administration mode. Measurement errors may also originate from choices on the questionnaire structure (e.g. the format for response scales), but this has been shown to be unrelated to the interview mode [24].

The remaining differences can be classified into three non-independent categories, interviewer impact, media-related effects, and information transmission [24, 25]. The presence of the interviewer in telephone surveys may generate higher sensitivity (social desirability) biases relative to web surveys [26]. Media-related effects strictly refer to the medium of communication used to administer the survey. These include a variety of elements, for example the locus of control (e.g. who controls the time of the day when the questionnaire is taken, its duration, etc.) which lies with the interviewer in telephone surveys, and with the respondent in web surveys. Different familiarity with the administration medium (in turn linked to attitudes towards technology) is another likely driver of heterogeneity in responses [27]. Information transmission refers to potential differences in how the same item is conveyed through different modes, e.g. verbal communication of response categories in telephone interviewing versus visualization in web-surveys [25]. Responses may vary depending on the web design of the questionnaire (e.g. number of items per page, layout, response categories) or the length of the telephone interview [27]. More specifically, there is evidence suggesting that telephone respondents are more likely to choose the positive endpoint rating compared to web respondents [24], a result which extends previous findings that aural respondents tend to give more positive answers than their mail counterpart [26].

The expectation that web surveys are less exposed to social desirability and sensitivity biases is confirmed by experimental results [28]. There is evidence that–after conditioning on internet use–responses within batteries of questions by telephone respondents are more heterogeneous than those provided by web survey respondents [4]. This evidence is even stronger when web surveys responses are benchmarked against face-to-face interviewees. The on-line mode is more exposed to satisficing behaviors, and results in less differentiation, a higher proportion of ‘don’t know’ answers, and higher non-response rates to individual items [29]. Another study [30] looked at satisficing behaviors related to order bias and primacy effects (i.e. very fast completion of the survey) in web surveys, and showed a relation between satisficing effects and low education.

## Study design and data

### Previous studies on support for healthy eating policies

The measurement of public support for healthy eating policy actions has received attention in the US and elsewhere, since the seminal study by Oliver and Lee [31], which explored the drivers of policy acceptance based on a CATI-RDD survey on a sample of 909 adults in 2001. Other RDD-based studies in the same area include Evans et al. [32] for the US, and Sikorski et al. [33] and Hilbert, Rief, and Braehler [34] for Europe. More recently, similar research questions were addressed in the US using on-line surveys [35–37]. In these works, the samples of respondents were extracted from on-line panels built probabilistically via RDD. Although these latter studies refer to their CATI predecessors and provide comparisons of results, no consideration has been given to the role of potential mode effects.

### Study design

The data for this specific two-country/two-mode study were collected as part of a wider international research project on the economic evaluation of healthy eating policies [38]. The project included a larger scale web survey conducted in five countries, aimed at eliciting public acceptance of policies to promote healthy eating and prevent obesity [39]. The questionnaire was designed by scientists within the research consortium, and the piloting and field work were run by GfK NOP Social Research.

The two-mode survey (CAWI and CATI) was conducted in Italy and the UK with the main purpose of identifying and quantifying the measurement and selection biases associated with running a web survey using a proprietary panel, a common approach in this area of research. The choice of these two countries among the five of the larger scale survey (also run in Belgium, Denmark and Poland), was driven by manifest differences in both food culture and internet penetration. The questionnaire was adapted from the one used in the larger scale web survey to meet the requirements of both administration modes and to reduce to the maximum possible extent any questionnaire-related measurement effect. The script for both surveys was written using a multi-modal data collection package. The English version of the script was checked by researchers to confirm that question wording and routing instructions were as required. For the Italian version, a back translation of both the questionnaire items and instructions ensured consistency of the contents. The sampling frame for the CAWI survey was provided by the GfK NOP e-panel, which–at the time of the survey–included about 236,000 contacts in the UK and 73,000 in Italy. The e-panel is built on a voluntary basis whereby panelists sign up online through ads, websites, social forums, etc. Selected respondents received an invitation with details of the research and an unique link to access the questionnaire.

Field work was conducted between 7 and 22 February 2011. Ethical clearance was obtained from the project lead institution’s (University of Reading) ethics board. Informed consent by participants was obtained by the research agency running the field work, electronically for the CAWI survey and verbally for the RDD CATI survey. The final sample sizes were 249 for the UK CATI, 251 for the Italian CAWI, and 250 for the other two surveys. Sampling was based on probabilistic methods, through stratified sampling (by age and gender) on the list of e-panelists for the CAWI survey, and RDD for the CATI survey. Replacements for non-respondents in the CAWI surveys were made by extracting units from the same stratum, while the CATI survey proceeded until the desired number of respondents was achieved. No quotas were applied.

The CATI sampling strategy followed a RDD approach along with the Rizzo Brick variant of the next birthday rule [40] for selecting individual respondents within the contacted household. In both countries interviewing was conducted in the evenings during the week and throughout the day at weekends. At least three call-backs at different days of the week and different times of the day were planned to address missing contacts and non-response, but in many cases interviewers made more than three call-backs to achieve an interview or another final outcome. The average duration of the interview was 15.5 minutes in UK and about 17.5 minutes in Italy. Depending on the commonly accepted definition of response rate by the American Association for Public Opinion Research [41], CATI response rates ranged between 3% in both countries (AAPOR RR1) and 9.4% in the UK or 8% in Italy (AAPOR RR4). Proper response rates for the CAWI survey cannot be computed, because there is no information on the selection process for the opt-in panel, thus we can refer to participation rates as the percentage of completed questionnaires on the total number of invitees from the GfK NOP e-panel, which were 15% in the UK and 27% in Italy.

### The questionnaire

The questionnaire included 27 questions, building on and extending the questionnaire by Oliver and Lee [31]. The questionnaire was identical for the CAWI and CATI surveys, with the exception of a filtering question specific to the CATI to identify internet users. Only internet users in the CATI were administered a subsequent question on the frequency of internet use (hours actively spent on the internet during the previous week). The questionnaire included other questions to elicit the characteristics of respondents (demographics, household composition, subjective health status, education level, internet use, levels of physical activity according to the IPAQ classification, financial conditions, level of food expenditure), some eating habits, and perceived risks to own health. The measurement of public support for healthy eating policies was based on agreement with 20 statements (Table 1), measured on a 5-point Likert scale and including a ‘don’t know’ (DK) option, while other forms of non-response were not allowed for. The order of items was subject to random rotation to avoid order biases. Since the main objective of the study was to produce a synthetic ranking of the policy options, a reclassification of the policy acceptance items into three levels (supportive, neutral, not supportive) was adopted in subsequent analysis. Respondents were classified as supportive if they agreed or strongly agreed to the policy statement, as opponents if they disagreed or strongly disagreed, while neutral responses and DKs were maintained as in the original classification. Support rates were intended as the proportion of supportive respondents on the total number of respondents, including ‘don’t knows’. The number of respondents who chose the DK option varied across items, ranging between none (for the EDUSCHOOL item in the UK CAWI survey) and 19 (i.e. 7.6%, for the FATTAX item in the Italian CAWI survey). On average the rate of DKs was lower in the UK (and for CAWI surveys), with 2.1% of responses (2.7% in the CATI) relative to 3% in Italy (3.3% for the CATI).

**Table 1:** List of items measuring support for healthy eating policies.

Policy support item	Short name
The government should ban advertising for junk food and fast food that is aimed at children	ADVBANCHILD
The government should ban advertising for junk food and fast food that is aimed at adults	ADVBANADULT
The government should spend money for information campaigns informing people about the risks of unhealthy eating	SOCIALMKTG
Education to promote healthy eating should be provided in all schools	EDUSCHOOL
The government should subsidise firms which provide programmes to train their employees in healthy eating	EDUWORK
All foods should be required to carry labels with calorie and nutrient information	LABELING
All restaurants should be required to provide calorie and nutrient information in menus	MENUS
The food industry should cooperate in financing governmental campaigns that promote healthy eating	INDCOOPER
The government should award companies for healthy food innovations	INDAWARDS
The government should impose taxes on unhealthy food and use the proceeds to promote healthier eating	FATTAX
The government should subsidise fruit and vegetables to promote healthier eating	THINSUBS
The government should provide vouchers to low-income families to buy healthy foods at reduced prices	VOUCHERS
Vending machines should be banned from our schools	VENDBAN
The government should regulate the nutritional content of school meals	SCHOOLMEAL
The government should regulate the nutritional content of workplace meals	WORKMEAL
The government should work with the food companies to improve the nutritional content of processed foods	VOLUNTSTD
The government should impose on food companies limits on certain ingredients to improve the nutritional content of processed foods	COMPSTD
TV-stations should give free air-time to governmental campaigns that promote healthier eating	FREEADS
There should be public measures like free home delivery to support easier access to healthy foods for the elderly and those with lower incomes	ACCESS
VAT rates should be lower for healthy foods and higher for unhealthy foods	VAT

### Characteristics of respondents

As shown in Table 2, there are major differences in the characteristics of respondents, both between countries and between the two survey modes. Those participating to the CAWI survey are on average younger, wealthier and with a higher education level, consistently with most existing evidence on the digital divide [1, 8, 20]. In general, the differences between the two modes are larger in Italy, where the internet penetration rate is lower. Country disparities are also large. The proportion of male respondents was higher in the UK, especially in the CAWI mode (62.8% in UK compared to 35.5% in Italy). The main differences are found in the health status and eating habit variables. The body mass index of UK respondents, computed from self-reported height and weight, was higher (and above the overweight threshold of 25). Italians tend to report a higher risk perception for all health risks, with averages always above 4 (neutrality in the 7-point scale), while in the UK only the ‘own weight’ risk factor is perceived as (slightly) serious. Interestingly, the CAWI-CATI gap goes in opposite directions across the two countries when considering weight and dietary risks. In Italy, the average risk perception is higher for CATI respondents, whereas in the UK those responding to the CAWI survey perceive a higher risk. The prevalence of self-reported health conditions is higher in the UK than Italy, with the exception of cholesterol. As for risk perception, the rates of diagnosed conditions for CATI respondents in Italy are much higher than their CAWI counterparts, whereas the UK differences are small. Italy and the UK are also known to show major differences in terms of food culture [42] and healthy eating policy [43] and these are reflected in the eating habit variables. Italians eat out more frequently, whereas the consumption of pre-packaged and prepared meals is more common in the UK, although this difference is negligible for CAWI respondents.

**Table 2:** Mean characteristics of CAWI and CATI respondents, by country.

		UK				Italy			
Variable	Measurement Unit	CAWI		CATI		CAWI		CATI	
Age of respondent	Years	53.36	(13.56)	55.05	(16.82)	37.68	(12.10)	51.48	(15.90)
Household size		2.47	(1.25)	2.15	(1.31)	3.09	(1.20)	2.74	(1.31)
Children <16 in the household	%	20.4		22.5		30.3		26.8	
Single respondent	%	25.2		24.1		43.4		18.8	
Male respondent	%	62.8		39.4		35.5		29.2	
Low education	%	17.7		29.3		9.3		30.5	
Medium education	%	33.8		34.9		60.6		44.2	
High education	%	48.5		35.8		30.1		25.3	
Body-mass index	Kg/m^2^	27.09	(4.48)	25.94	(5.41)	23.86	(4.49)	24.52	(4.11)
Perceived risk from:									
own weight	1 = Not at all serious; 7 = Very serious	4.32	(1.75)	3.69	(1.97)	5.12	(1.66)	5.40	(1.61)
own eating habits		3.91	(1.59)	3.57	(1.90)	5.10	(1.60)	5.70	(1.38)
pollution		3.04	(1.61)	2.48	(1.75)	4.97	(1.73)	4.77	(2.28)
own stress level		3.84	(1.75)	3.55	(1.94)	5.24	(1.58)	4.79	(1.88)
Financial condition of the household	1 = manage very well; 5 = severe difficulties	2.82	(1.10)	2.55	(1.05)	3.31	(0.98)	2.81	(0.88)
Health status	1 = Very bad; 5 = Very good	3.52	(0.89)	3.93	(1.04)	3.78	(0.74)	3.71	(0.76)
High blood pressure	%	32.4		32.9		16.7		24.8	
High blood cholesterol	%	29.6		26.1		18.3		26.8	
Heart disease	%	6.8		8.8		6.4		7.6	
Diabetes	%	8.4		9.6		8.0		6.4	
Other health conditions	%	21.2		25.3		15.5		16.4	
Food expenditure (HH)	€/week/per capita	74.32	(36.62)	72.21	(37.48)	25.69	(13.54)	33.84	(20.37)
Eating habits									
Eating out at lunch	Times/ week	1.07	(1.49)	1.05	(1.48)	1.51	(1.78)	1.62	(2.16)
Eating out at dinner		0.51	(0.53)	0.65	(0.97)	0.93	(0.90)	0.71	(1.05)
Fast food restaurants		0.28	(0.42)	0.28	(0.46)	0.60	(0.98)	0.29	(0.82)
Pre-packaged/ prepared meals		0.59	(1.01)	0.77	(1.22)	0.55	(1.02)	0.21	(0.55)
Physical activity	1 = No activity;4 = Intense	2.67	(1.14)	2.96	(1.03)	2.83	(1.12)	2.73	(1.05)
Access internet at work/university	%	33.2		46.4		41.0		31.6	
Frequency of internet use	Hours per week	19.60	(12.39)	10.65	(11.75)	19.15	(12.96)	8.18	(10.38)
Internet user	%	100.0		72.5		100.0		64.8	
*Sample size*		*250*		*249*		*251*		*250*	

Beyond cultural and lifestyle differences, there is a major disparity in internet coverage rates, intended as the proportion of individuals who have accessed the internet at least once over the last 12 months, 87% in the UK and 59% in Italy according to 2011 Eurostat data. Estimates from the CATI samples are different and smaller (72.5% in the UK, 64.8% in Italy), but together with the frequency of internet use they still reflect a substantially higher penetration in the UK.

The heterogeneity found across observed respondent characteristics provides the rationale for this study. Clearly, estimates of support rates for healthy eating policies between the two administration modes are hardly comparable without some balancing of these variables.

## Methods

### Testing strategy and assumptions

The study provides information on the following groups of respondents: (a) respondents from the CAWI survey, who are obviously internet users; (b) respondents from the CATI survey who are internet users (CATI_INT); (c) respondents from the CATI who are not internet users (CATI_NOINT). This design enables us to isolate selection and measurement effects, based on few assumptions and the application of propensity score matching techniques to make these groups comparable, conditional on a selected set of characteristics. The overall mode effect, intended as the different outcome between the CAWI and CATI surveys, is a combination of a measurement effect and a selection effect.

First, we consider the measurement effect. An experimental estimate of the measurement effect would require the same respondents to answer to both the CATI and CAWI questionnaires. We approximate such ideal situation by comparing CAWI and CATI_INT respondents after conditioning on a set of observed respondent characteristics (*X*), and their frequency of internet use (*INTERNET*). For individuals who have similar characteristics, and similar frequency of internet use, we ascribe the average difference in outcomes to the gross measurement effect (*GME*), intended as the difference in measurement errors:

$$GME=E(Y_{CAWI}|X,INTERNET)-E(Y_{CATI\_INT}|X,INTERNET)$$

This estimate of the measurement effect is gross of a residual selection effect, if there exists a set of unobservable respondent characteristics (*U*) that are not captured by the observed characteristics *X* and are relevant in explaining different responses across the two modes. An implicit assumption is that this measurement effect would not be different if we could measure it on non-internet users, so that information on internet users is sufficient to estimate the *GME*.

Second, we explore the selection effect associated with internet use (*SE*). Consistently with the classification of survey errors adopted in this paper, we attribute this selection effect to a coverage component (differences in the sampling frames) and to non-response behaviors which differ across the two modes. We only refer to the CATI sample to isolate this effect, so that in a single mode there is no measurement effect. This means that we implicitly assume that the selection effect would not be different if we could measure it on the CAWI sample, which is impossible because it excludes non-internet users. Under this assumption, the selection effect is:

$$SE=E(Y_{CATI\_INT})-E(Y_{CATI\_NOINT})$$

Consistently with our objectives and evidence from the literature, we make a further decomposition of the causes of *SE*, identifying two broad categories of selection error, one generated by the selection bias on observed variables *X* (the fact that internet non-users are potentially different from internet users in terms of these characteristics), and one generated by the fact that a further selection bias occurs on unobserved characteristics, for example attitudes towards technology which may lead two otherwise identical individuals to make different choices on whether to use the internet or not. If these unobserved characteristics are related to the outcome variable, then they will add to the selection bias. If we indicate with *SE*_*OBS*_ and *SE*_*UNOBS*_ these two components of selection effect, then *SE* = *SE*_*OBS*_ + *SE*_*UNOBS*_ and an estimate of *SE*_*UNOBS*_ can be obtained by looking at the different outcomes between internet users and non-users conditional on the observed characteristics *X* and, as before, using only data from the CATI sample to exclude measurement effects:

$$SE_{UNOBS}=E(Y_{CATI\_INT}|X)-E(Y_{CATI\_NOINT}|X)$$

### Propensity score matching

A common post-stratification procedure to adjust for selection bias in web surveys consists in weighting observations using propensity scores (PSW). Propensity scores reflect the probabilities of being a web survey respondent conditional on a set of observed respondent characteristics (which we call *X*, as before). Their estimates are obtained as predicted probabilities from a binary regression model, where the selection variable is regressed upon these characteristics, using data from a reference survey which is assumed to be unaffected by selection errors, or simply more representative of the target population. There are several applications to adjust estimates from web surveys [6, 11, 44, 45]. Alternatively, propensity scores are used to match observations from two samples which differ in terms of *X* because of imperfect (or absent) randomization (PSM), as originally conceived by Rosenbaum and Rubin [46]. In both methods, the application of propensity scores balances the observed covariates *X*, but not necessarily the unobserved covariates *U*, unless they are correlated with *X* [47]. It follows that PSM is only successful when *X* captures all covariate effects on the outcome other than the experimental effect being investigated, and there is no selection on unobservables. This is the conditional independence assumption (CIA), also known as ignorability, as it allows unobserved variables *U* to be ignored [48]. The CIA condition also requires the variables in *X* to be unaffected by the experimental effect (e.g. the interview mode or the use of internet). In other words, the variables on the right-hand side of the binary regression model must be exogenous to avoid introducing further biases. Hence, the availability and the choice of the variables to be included in *X* is crucial to perform a meaningful PSW or PSM to control for selection biases in internet surveys [49]. Thus, any variable whose measurement is influenced by the survey mode (for *GME*) or which is affected by internet use (for *SE*_*UNOBS*_) should not be used as a covariate in the probit model. This generates a trade-off between considering as many covariates as possible, and including only those which can be safely assumed to be exogenous.

Based on the propensity score estimates, the comparison of the average outcome between the matched samples may be based on different algorithms, depending on the units being matched (one-to-one, one-to-several, several-to-several) and on the matching criterion used (e.g. nearest unit, within a radius, etc.), see [50] for an overview. Here we present results only from the stratification matching algorithm, but the findings were robust to the application of four different algorithms, which produced very similar estimates.

Stratification consists in grouping individuals in each sample into intervals (strata) based on their propensity score values, where the number and size of the strata is determined in a way to meet the so-called balancing property (BP). Meeting this property implies that within each stratum the average values of the covariates are not statistically different between the two surveys. Stratification was proposed as a method to control for selection bias well before PSM [51], and [52] first applied it to propensity scores using quintiles. More sophisticated stratification strategies have been developed to set the number and size of the strata while meeting the BP [53]. We follow the algorithm proposed by Becker and Ichino [54], which starts by subdividing the propensity scores into five equally spaced intervals, then tests the null hypothesis of equal mean propensity scores across the two samples. Strata where this hypothesis is rejected are split in halves, and the algorithm proceeds until there is no rejection. At this stage, a further mean comparison test on the covariates *X* is needed to ensure the necessary condition for the BP. The algorithm might fail to find a stratification which meets the BP, in which a less parsimonious specification of the binary regression is needed.

For the purpose of our study, we adopt stratification matching first to isolate the *GME* when comparing outcomes from the CAWI (target) and CATI (benchmark) surveys, then to estimate the selection effects on unobservables (*SE*_*UNOBS*_) between internet users (target) and non-users (benchmark) from the CATI survey. The procedure we implement consists in three steps: (1) estimate a probit model to obtain propensity scores, where the dependent variable is *D* = 1 if the respondent belongs to the target group and *D* = 0 for the benchmark group; (2) apply the stratification matching algorithm to the estimated propensity scores; (3) estimate the average difference in outcomes, which consists in a weighted average of the differences for each stratum, where the weight is the relative frequency of target observations. The procedure to estimate *GME* and *SE*_*UNOBS*_ is run separately for Italy and the UK, hence our PSM strategy involves the estimation of four probit models.

The set of potential covariates is the list of variables in Table 2. In matching the CAWI and CATI samples to estimate the *GME*, some of the covariates are potentially affected by the interview mode themselves, and including them could violate the CIA requirement. Thus, our probit estimates exclude those variables related to health status, weight, eating out habits, physical activity, and financial conditions based on the rationale that these self-reported measurements are themselves likely to be exposed to an interviewer effect. However, as a robustness check, we explored the effects of including all the covariates regardless of the risk of endogeneity, and the difference in the *GME* estimates was negligible.

## Results

The rates of support for each policy item are reported in Table 3, together with the outcome of a t-test on the differences between the CAWI and CATI estimates. With only three exceptions out of 40 comparisons, support rates are significantly different at the 5% significance levels.

**Table 3:** Rates of support by administration mode and country.

	UK				Italy			
Outcome variable	CAWI	CATI	Difference^a^		CAWI	CATI	Difference^a^	
	(1)	(2)	(1)-(2)		(1)	(2)	(1)-(2)	
ADVBANCHILD	0.71	0.83	-0.12	***	0.57	0.82	-0.25	***
ADVBANADULT	0.47	0.57	-0.10	**	0.47	0.68	-0.21	***
SOCIALMKTG	0.52	0.70	-0.18	***	0.77	0.90	-0.13	***
EDUSCHOOL	0.86	0.95	-0.10	***	0.82	0.98	-0.16	***
EDUWORK	0.30	0.52	-0.22	***	0.56	0.65	-0.09	**
LABELING	0.72	0.90	-0.17	***	0.84	0.96	-0.12	***
MENUS	0.50	0.55	-0.04		0.58	0.64	-0.06	
INDCOOPER	0.63	0.78	-0.15	***	0.75	0.90	-0.15	***
INDAWARDS	0.53	0.69	-0.15	***	0.73	0.85	-0.12	***
FATTAX	0.44	0.60	-0.16	***	0.54	0.64	-0.11	**
THINSUBS	0.58	0.69	-0.11	***	0.77	0.84	-0.08	**
VOUCHERS	0.50	0.68	-0.18	***	0.75	0.82	-0.08	**
VENDBAN	0.54	0.69	-0.15	***	0.42	0.60	-0.17	***
SCHOOLMEAL	0.66	0.86	-0.20	***	0.75	0.91	-0.16	***
WORKMEAL	0.22	0.36	-0.14	***	0.70	0.76	-0.06	
VOLUNTSTD	0.66	0.81	-0.15	***	0.74	0.88	-0.14	***
COMPSTD	0.58	0.78	-0.20	***	0.71	0.87	-0.15	***
FREEADS	0.53	0.68	-0.15	***	0.73	0.90	-0.17	***
ACCESS	0.53	0.73	-0.20	***	0.71	0.87	-0.16	***
VAT	0.58	0.78	-0.20	***	0.60	0.69	-0.09	**
*Sample sizes*	*250*	*249*	** **		*251*	*250*		

Notes: Rates of support are computed as the ratio between the number of those who agreed/strongly agreed to the policy statement and the total number of respondents including those who chose the ‘don’t know’ option.

^a^ Asterisks refer to significance levels from a mean comparison t-test assuming unequal variances

*** = 0.01 s.l.

** = 0.05 s.l.

For most items, there are also large country differences. For example, only 22% of UK CAWI respondents support regulations on workplace meals, against 70% in the Italian CAWI sample. These differences also vary by mode. The CAWI support rate for banning advertising to children is 71% in the UK and only 57% in Italy, whereas the difference is negligible (83% vs. 82%) in the CATI sample.

First, we estimate the *GME* to explore to what extent these differences can be explained by a measurement effect. We compare the CAWI sample with the CATI_INT subgroup of internet users from the CATI sample. While both groups include internet users only, diverging estimates of support rates are likely to stem from other differences in the characteristics of respondents. Our PSM strategy involves matching these two groups on the observed covariates, in order to mitigate the selection effect generated by the different sampling frames and non-response rates. The inclusion of covariates measuring internet behaviors (frequency of use, where it is accessed) aims at balancing other unobserved variables that are relevant to entering an e-panel. We cannot rule out that residual selection effects on unobservable characteristics affect the comparison between the matched CAWI and CATI_INT samples, but this is the best possible approximation of the pure measurement effect.

Second, using CATI data only, we explore the selection effect associated with being an internet user by comparing the CATI_INT and CATI_NOINT sub-samples. The simple difference between the outcomes is a raw estimate of the overall selection effect (*SE*) associated with internet use. Then, we isolate that component of *SE* which cannot be ascribed to differences in the observed covariates. PSM on the two CATI sub-samples balances the observed characteristics of internet users and internet non-users, so that any remaining difference in outcomes can be ascribed to differences in any unobserved respondent characteristic not captured by the observed covariates.

Probit estimates are shown in Table A in S1 Appendix. The backward selection procedure was aimed at maintaining all those variables with a significance level below 0.20, but only one variable (education in Italy) exceeds the 0.10 threshold. The goodness-of-fit of the *GME* probit model is acceptable (UK 0.26; Italy 0.31 for Italy). The second pair of probit models is functional to the estimation of *SE*_*UNOBS*_ and propensity scores are expected to balance the characteristics of the CATI_INT and CATI_NOINT sub-samples. The goodness of fit is good (UK 0.57; Italy 0.48) and the marginal effects confirm the role of age and education, while the financial condition variable is non-significant. Other significant covariates are related to risk perception and health (especially in the UK), and to the BMI in Italy.

A summary of the estimated differences is provided in Table 4, which reports median values across the 20 policy items. A more detailed analysis of support rates by individual policy item is provided in Table B in S1 Appendix. The first column shows the raw median difference between CAWI and CATI responses. In both countries, CAWI support rates are significantly lower (-15.3% in the UK and -13.5% in Italy), and there is a corresponding positive shift in the median rate of neutral respondents. We also observe a relatively small reduction in the proportion of opponents (only significant for Italy, -0.9%) and a 1% median increase in ‘don’t know’ answers in the UK.

**Table 4:** Difference in median rates across the policy items, *GME* and *SE*_*UNOBS*_ estimates.

	UK									
	*CAWI-CATI*		*CAWI-CATI*_*INT*_		*GME*		*CATI*_*INT*_*-CATI*_*NOINT*_		*SE*_*UNOBS*_	
SUPPORT	-0.153	***	-0.134	***	-0.088	***	-0.103	***	-0.190	***
NEUTRAL	0.150	***	0.138	***	0.132	***	0.038	***	0.067	***
OPPONENT	-0.001		-0.017	**	-0.029	**	0.081	***	0.105	***
DON'T KNOW	0.010	*	0.012	**	0.011	*	-0.003		0.010	
	Italy									
SUPPORT	-0.135	***	-0.116	***	-0.102	***	-0.064	***	-0.031	**
NEUTRAL	0.143	***	0.138	***	0.132	***	0.029	***	0.036	***
OPPONENT	-0.009	**	-0.027	***	-0.030		0.041	***	-0.030	
DON'T KNOW	0.004	^ ^	0.013	^ ^	0.008	^ ^	-0.013	**	0.012	**

Asterisks refer to significance levels from a Wilcoxon signed-rank test on the null hypothesis of median equal to 0

*** = 0.01 s.l.

** = 0.05 s.l.

* = 0.10 s.l.

The second column–where the CAWI-CATI comparison is restricted to internet users–confirms and reinforces these findings. The negative shift in those opposing the policy statements becomes larger (UK -1.7%, Italy -2.7%) and significant. The third column is the *GME* estimate, hence what is left after matching on the observed covariates is ascribed to the mode-related measurement effect. The evidence is strong and consistent across the two countries and shows that CAWI respondents are less likely to state support and more likely to state neutrality than their CATI counterpart. As shown in Table B in S1 Appendix, item-specific results are variable in terms of magnitude, but strongly consistent with a reduction in support rates, which can be as large as 25% for items like changing VAT rates in the UK or imposing advertising bans in Italy. These results confirm the previous finding that respondents of telephone surveys are more prone to ‘take side’.

The last two columns of Table 4 explore the median selection effect associated with internet use. The raw difference between users and non-users is again similar across the two countries. Even when considering a single administration mode, internet users are less likely to be supportive of healthy eating policies (UK -10.3%, Italy -6.4%). The shift, however, does not only occur towards neutrality (UK +3.8%, Italy +2.9%), but to a larger and significant extent towards opposition (+8.1% in the UK, +4.1% in Italy). Once the matching algorithm is applied, the estimate of *SE*_*UNOBS*_ (last column of Table 4) shows the residual difference. The UK results are striking. After matching, the distance in support rates becomes larger (a median of -19% and negative values for all policy items, see Table B in S1 Appendix), and the consequent increase in the proportion of neutrals (+6.7%) and opponents (+10.5%) is also larger and highly significant. We also find a residual reduction in support rates and an increase in neutrality rates in Italy, but much smaller (-3.1% and +3.6%, respectively). In other words, matching on observed covariates makes internet user and non-users more similar in terms of support in Italy, whereas the difference between the two groups becomes more conspicuous in the UK, indicating a major role for unobservable characteristics associated with internet use.

We can only speculate about the determinants of this difference, for example those with an easier access to technology and information may be less inclined to accept paternalism, and more confident about individual abilities to adopt appropriate eating behaviors. The finding of a less pronounced effect in Italy is consistent with a much lower proportion of internet users compared to the UK. As internet access becomes widespread, being an internet user or not becomes a matter of individual choice and specific unobserved characteristics. Among these, together with attitudinal factors, we could include potential infrastructural gaps. As the proportion of non-users narrows down, infrastructural gaps are less likely to occur and we are more likely to capture attitudinal differences, even if the sample against which users are matched becomes smaller.

## Discussion and conclusion

Our PSM strategy on a two-country/two-modes survey on stated support to a variety of healthy eating policy interventions leads to the following main conclusions:

(a) Estimates of support are highly dependent on the survey mode, and discrepancies between a CAWI probabilistic sample from a proprietary panel, and a CATI-RDD sample can be as large as 25% for some items
(b) Our estimate of the mode-related measurement effect suggests that the CAWI mode systematically leads to higher neutrality rates relative to CATI;
(c) The increase in CAWI neutrality rates stems from relatively lower support rates and–to a lower extent–lower opposition rates;
(d) Our estimate of the selection effect associated to internet use also suggests that internet users are less likely to support policy interventions relative to non-users;
(e) This selection effect is amplified in the UK, where the internet access rate is higher. As internet coverage increases, the gap between users and non-users is smaller in terms of observed characteristics, but the selection factor which depends on unobservable (or unobserved) characteristics may become more prominent.
(f) In relation to the results reported in [39], our analysis provides evidence that the mode effect is hardly influential for opposition rates, and relevant in shifting responses from support to neutrality. Hence, the findings discussed in the study remain valid, although support rates can be considered as a lower benchmark. Given the consistency of this underestimation of support rates across the policy support items, the quantitative exploration of the determinants of support in [39] is unlikely to be affected by the bias.

Our study is subject to various limitations intrinsic to its empirical nature, and requires some simplifying assumptions. First, our estimate of measurement effect is also gross of a selection effect which can be traced back to unobserved characteristics. Second, we are unable to isolate the non-response component of the selection effect. Furthermore, our CAWI sample is randomly extracted from a self-selected proprietary panel. The effectiveness of our matching procedure in mitigating the impact of self-selection into the sampling frame depends on the range of available covariates, but a purely probabilistic CAWI sample may return a lower measurement error.

Our results might be refined by a variety of ad-hoc studies. More specifically, it would be valuable to obtain a more explicit assessment of the selection effect induced by the use of the internet as distinguished by the overall mode effect. For example, an experiment administering a web survey to non-internet users would allow to validate our estimates that are based on CATI respondents only. Similarly, it could be interesting to extend our analysis to consider face-to-face interviews, which would also allow to have a more explicit estimate of the interviewer effect as a separate source of bias from the medium and information effects. Other important extensions would be the collection of information to explore the relevance of the non-response dimension and the impact of extracting the CAWI sample from a proprietary panel. These analyses would shed further light on the overall representativeness of CAWI estimates relative to the target populations. While future research might address these limitations, the strength and consistency of our estimates across two countries and 20 different policy items provides a good degree of confidence in the above listed conclusions. The relevance of the measurement effect is hardly surprising or innovative, and our contribution is simply to suggest a procedure to estimate its magnitude. Instead, our results pinpoint the risks of ignoring the portion of selection error which does not depend on differences in the observed characteristics between internet users and non-users. One might wrongly assume that the potential biases associated with internet surveys fade out as internet coverage increases, especially after controlling for demographic and socio-economic differences. Instead, we claim that higher internet access is associated with larger differences in factors which are not necessarily measured, for example the attitude towards technology, information processing skills and opportunities associated with internet access, subjective health status or risk perception. When–as in our case–these factors are likely to impact on the variables of interest, web surveys not adopting countermeasures are subject to potentially serious biases even after post-stratification weighting.

## Supporting information

S1 Appendix Additional estimation results. Probit models for the propensity score models by country and PSM-based Gross Measurement Effects and Selection Effect on Unobservables.
(DOCX)

S1 Dataset SPSS data. (SAV)
