# Supplementary material for: Estimation of unobservable selection effects in on-line surveys through propensity score matching: An application to public acceptance of healthy eating policies
Source: PLoS One. 2018 Apr 17;13(4):e0196020. doi: 10.1371/journal.pone.0196020 (PMC5903641; doi:10.1371/journal.pone.0196020)
Supplement: S1 Appendix — Probit models for the propensity score models by country and PSM-based Gross Measurement Effects and Selection Effect on Unobservables. (DOCX) [file pone.0196020.s001.docx]

**S1 – APPENDIX**

**Table A. Probit models for propensity score matching, by country**

|  | Dependent variable | | | | | | | |
| --- | --- | --- | --- | --- | --- | --- | --- | --- |
|  | CAWI=1 vs CATI=0 (*GME*) | | | | CATI_INT_=1 vs CATI_NOINT_=0 (*SE_UNOBS_* ) | | | |
|  | UK | | Italy | | UK | | Italy | |
|  | Marginal effect | | Marginal effect | | Marginal effect | | Marginal effect | |
|  | (p-value) | | (p-value) | | (p-value) | | (p-value) | |
| Age of respondent | 0.005 | (0.034) |  |  | -0.007 | (0.000) | -0.019 | (0.000) |
| Body-mass index |  |  |  |  |  |  | -0.022 | (0.007) |
| Household size | 0.089 | (0.001) | -0.048 | (0.088) |  |  |  |  |
| Food expenditure of the household |  |  | -0.007 | (0.001) |  |  |  |  |
| Perceived risk from own weight | 0.032 | (0.032) |  |  |  |  |  |  |
| Perceived risk from own eating habits |  |  | -0.073 | (0.001) | 0.013 | (0.070) |  |  |
| Perceived risk from pollution | 0.037 | (0.027) |  |  |  |  |  |  |
| Perceived risk from own stress level |  |  | 0.030 | (0.125) | 0.021 | (0.010) |  |  |
| Children <16 in the household | -0.249 | (0.003) | 0.126 | (0.087) | 0.089 | (0.041) |  |  |
| Single respondent |  |  | 0.179 | (0.005) |  |  |  |  |
| Male respondent | 0.169 | (0.002) | -0.134 | (0.032) |  |  | 0.207 | (0.002) |
| Education level |  |  | -0.059 | (0.193) | 0.066 | (0.000) | 0.303 | (0.000) |
| Frequency of internet use | 0.122 | (0.000) | 0.141 | (0.000) |  |  |  |  |
| Access internet at work/University | -0.133 | (0.029) | -0.131 | (0.023) |  |  |  |  |
| Health status of respondent |  |  |  |  | 0.038 | (0.009) |  |  |
| Heart disease |  |  |  |  | 0.055 | (0.001) |  |  |
| Diabetes |  |  |  |  | -0.597 | (0.001) | 0.149 | (0.097) |
| Other health condition |  |  |  |  | -0.086 | (0.023) |  |  |
| Eating out at dinner | -0.095 | (0.012) |  |  |  |  |  |  |
| Eating in fast food restaurants |  |  |  |  | -0.052 | (0.018) |  |  |
| *Number of observations* | *425* | | *402* | | *227* | | *244* | |
| *McFadden Pseudo R^2^* | *0.26* | | *0.31* | | *0.57* | | *0.48* | |

**Table B. PSM estimates of** $\boldsymbol{GME}$ **and** $\boldsymbol{S}\boldsymbol{E}_{\boldsymbol{UNOBS}}$ **in support rates, by country and item**

|  | GME | | | | SE_UNOBS_ | | | |
| --- | --- | --- | --- | --- | --- | --- | --- | --- |
| Outcome variable | UK | | ITA | | UK | | ITA | |
| ADVBANCHILD | -0.069 |  | -0.246 | *** | -0.188 | *** | -0.021 |  |
| ADVBANADULT | -0.007 |  | -0.157 | * | -0.310 | *** | -0.010 |  |
| SOCIALMKTG | -0.085 |  | -0.102 | * | -0.283 | ** | 0.069 |  |
| EDUSCHOOL | -0.090 | *** | -0.160 | *** | -0.133 | ** | -0.025 | ** |
| EDUWORK | -0.136 | * | 0.074 |  | -0.317 | ** | -0.095 |  |
| LABELING | -0.138 | ** | -0.004 |  | -0.063 |  | -0.060 | *** |
| MENUS | 0.015 |  | 0.061 |  | -0.265 | ** | -0.036 |  |
| INDCOOPER | -0.082 |  | -0.222 | *** | -0.151 |  | -0.074 | *** |
| INDAWARDS | -0.067 |  | -0.046 |  | -0.192 |  | -0.017 |  |
| FATTAX | -0.163 | ** | -0.016 |  | -0.284 | ** | -0.045 |  |
| THINSUBS | -0.076 |  | -0.147 | *** | -0.260 | *** | -0.002 |  |
| VOUCHERS | -0.130 | * | -0.061 |  | -0.175 |  | -0.168 | *** |
| VENDBAN | -0.111 |  | -0.102 |  | -0.050 |  | -0.102 |  |
| SCHOOLMEAL | -0.181 | *** | -0.159 | *** | -0.137 | * | 0.045 |  |
| WORKMEAL | -0.088 |  | -0.026 |  | -0.202 |  | -0.079 |  |
| VOLUNTSTD | -0.063 |  | -0.103 |  | -0.143 | ** | 0.006 |  |
| COMPSTD | -0.188 | *** | -0.108 |  | -0.060 |  | -0.150 | *** |
| FREEADS | 0.067 |  | -0.093 |  | -0.277 | ** | -0.111 | *** |
| ACCESS | -0.218 | *** | -0.161 | ** | -0.224 | ** | 0.037 |  |
| VAT | -0.249 | *** | -0.026 |  | -0.179 | *** | 0.160 |  |

*** = 0.01 significance level, ** = 0.05 s.l., * = 0.10 s.l.
